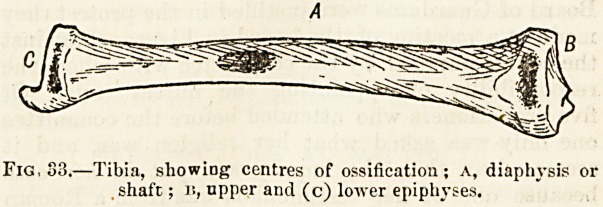# The Hospital. Nursing Section

**Published:** 1902-03-22

**Authors:** 


### The Hospital.

### Nursing Section.

Contributions for this Section of "The Hospital" should be addressed to the Editor, "The Hospital" Nursing Section, 28 & 29 Southampton Street, Strand, London, W.C.

No. 808.—Vol. XXXI. *SATURDAY, MARCH* 22, 1902.

### Notes on News from the Nursing World.

### THE ROYAL RED CROSS.

At the Investiture at St. James's Palace last week, the King conferred the decoration of the Royal Red Cross upon the following nurses :—Miss Agnes Mary Waterhouse, late of the Indian Nursing Service, who was attached to the military base near Tientsin ; Miss Martha Sarah Bidmead, a South Australian nursing sister ; and Miss E. Nixon, a New South Wales nursing sister, who were both attached to the hospital staff at Bloemfontein during the epidemic of typhoid soon after the outbreak of the war.

### THE ROYAL VISIT TO MANCHESTER.

The Nurses' Hostel, at Manchester, was the scene of a most picturesque gathering on the occasion of the Royal visit last week. By the kindness of the committee of the Manchester and Salford Sick Poor and Private Nursing Institution, a stand had been put up in front of the hostel for the district nurses to witness the procession. There were also a great number of private nurses from various institutions. Those resident in the hostel at the time gathered together on the porch and at the windows, their uniforms mingling prettily with the decorations on the house. When the procession came in sight the nurses threw off their cloaks, and stood to greet the Prince and Princess as they passed all too rapidly by.

### RYDE DISTRICT NURSING ASSOCIATION.

Princess Henry of Battenberg presided at the annual meeting of the Ryde District Nursing Association last week. The report which was adopted is of a very satisfactory character. Upwards of 7,000 visits were paid by the nurses, and 383 new cases were attended. The services of a fourth nurse were proved to be absolutely necessary in view of the increase of work, and Miss Lathleen was accordingly engaged. Among the several gifts of the year to the association was a bicycle for the use of the staff. The addition to the home built in memory of the founder of the institution, the late Dr. Alexander Davey, is now practically completed, and a friend having generously offered to furnish the new building, it will shortly be handed over to the committee. It was mentioned that the society has gained the highest possible commendation from the visiting inspector of Queen Victoria's Jubilee Institute, with which it is affiliated. The superintendent of the home, Miss Wyatt, had the honour of being presented to Princess Henry, who congratulated her upon the excellent work carried out by herself and her colleagues.

### THE BOMBAY GOVERNMENT AND NURSING.

We draw attention to the issue of an important resolution on grants-in-aid of nursing at Government hospitals in the Presidency of Bombay, which is given in another column. It shows that Lord Northcote, the Governor of the Presidency, is fully alive to the necessity of improving the nursing arrangement, and is prepared to render substantial help to the hospitals, which conform to the very reasonable conditions laid down. We trust, however, that the action now taken will not encourage the public in India to imagine that they are relieved from the moral responsibility of contributing adequately to the maintenance of the nursing staffs. The intention of the Bombay Government in voluntarily assuming an additional burden, is undoubtedly in order to supplement the proceeds of private subscriptions, and the policy adopted should have the effect of stimulating generosity, especially of the wealthy natives, who have not hitherto recognised their obligation in a satisfactory manner.

### THE ARMY NURSING RESERVE.

According to an official statement, the number of candidates for admission to the Army Nursing Service Reserve, whose applications had been examined to the end of last September, was nearly 1,500. Of these about 73 per cent. were accepted for service, and of those thus selected nearly 800 have served in South Africa, nearly 200 at military hospital stations at home, and a few at other places abroad. Out of the entire number employed only 10 have been dismissed, or called upon to resign. It is not stated whether these dismissals, or compulsory resignations, were due to inefficiency or misconduct. A few nurses have also resigned voluntarily. There have been 14 deaths in South Africa and two at other stations. It will be remembered that in the details we published of the conditions of admission to the Reserve, the limits of age were between 25 and 35, but the committee have found it necessary to extend this to 40 in the case of positions of unusual trust and responsibility.

### THE NAVAL CADET IN SICK QUARTERS.

On board the *Britannia*, at Dartmouth, South Devon, which has just been visited by the King and Queen, there are 240 cadets training to become officers in the Navy. If a slight case of illness occurs, the patient is nursed by male nurses, called sickbaymen, who have been trained in nursing in one of the naval hospitals. But if, after 48 hours, the patient is no better, he is sent ashore in a stretcher and taken in an ambulance to the sick quarters at Townstal, Dartmouth. This is a large house, furnished as a hospital for 17 cadets, and is at the summit of the hill, with a lovely view of the Dart and English Channel. At the front there is a sunny verandah with seats for the convalescents, and plenty of roses and flowers in the garden. There are two trained nurses living in the house, who are sisters belonging to Queen Alexandra's Naval Nursing Service. They do all the nursing required, and are on duty alternately. They also have night duty to do, if the cadets are seriously ill. Their meals are: breakfast at 8 A.M., dinner 12.30 P.M., tea 4 P.M., supper 7.30 P.M. Immediately after their meal, the cadets have theirs, which the sister on duty attends, and if there be many convalescents carves for them. The cadets are in bed at 8 P.M., and to each bed an electric bell is attached, so that they can summon the sister at any time during the night. A little further up the hill another house is rented by the Admiralty for cadets' infectious cases. Shortly, both these houses will be exchanged for a cadets' hospital, consisting of three separate blocks, two blocks for infectious cases only, and the other block for non-infectious cases.

### THE OPPONENTS OF WORKHOUSE NURSING REFORMS.

The admirable paper of Miss Gibson, matron of the Birmingham Infirmary, which appeared in our issue of last week *in extenso*, has, it appears, created a stir among the champions of workhouse masters and untrained matrons. Accepting Miss Gibson as the advocate of the nurses, for whom in her paper she claims that they should be subject only to the correction of the medical officer or the Guardians, the supporters of the present anomalous position affirm that they have no doubt that every effort will be made to establish a case before the Departmental Committee for abolishing the control which the master and matron of a workhouse now possess, and placing the nurses on a separate and independent footing. The masters are therefore warned to move in the matter without any further delay, "as otherwise they may find themselves inadequately prepared to grapple with their antagonists." However they may move, the masters will not, we imagine, be able to get the better of overwhelming evidence in favour of reform.

### CERTIFICATES OF WORKHOUSE INFIRMARIES.

At the Central Poor Law Conference, Mr. Humphreys, in concluding his paper, said that so far as nursing is concerned, the Welsh workhouse infirmaries are in a hopeless condition. The Mayor of Cardiff has since stated that the remark does not apply to the Union Infirmary in that prosperous port, and we do not suppose that Mr. Humphreys intended to include Cardiff. The Mayor went on to say that if he had been at the conference he should probably have referred to the existing method of certificating. At present, he said, a nurse received at the end of her probation a certificate signed by the chairman of the board of guardians, the chairman of the visiting committee, the clerk to the board, and the medical officer. He thought that a beneficial reform would be to have these certificates issued by the Local Government Board, "so that, instead of being more or less of a local character, they should have the authority attaching to anything issued by the Local Government Board." But what about the superintendent nurse? Her signature is of the utmost importance; in fact, without it the certificate should be of no value.

### THE ROYAL BRITISH NURSES' ASSOCIATION AND THE MIDWIVES BILL.

During the progress of the Midwives Bill through committee in Parliament Mr. T. P. O'Connor proposed to take from the Society of Apothecaries the right of selecting one of the medical practitioners to serve on the Central Midwives' Board, and to put in their place the Royal British Nurses' Association. In the discussion on the amendment, Mr. Heywood Johnstone said that midwifery was really not a substantial part of the work of the members of the Royal British Nurses' Association, though possibly one in ten of its members was a certificated nurse. Mr. O'Connor insisted on the right of the Royal British Nurses' Association to be represented, though Sir Michael Foster urged that the Bill dealt with midwives and not with nurses. It was said, Sir Michael remarked, that a physician was a surgeon and something more. So a midwife, though a nurse, was something more. Mr. O'Connor asked the committee to allow him to withdraw the amendment in order to enable him to raise the claims of the Royal British Nurses' Association later, but this the committee refused, and on a division the amendment was negatived by 23 votes to 5. When the clause was put as a whole Mr. O'Connor made an unsuccessful endeavour to procure its defeat. The Bill, as amended, has been reported to the House.

### TRAINING AT FIJI.

The Colonial Hospital at Suva, Fiji, is now recognised as a training school by the Royal British Nurses' Association, and by the Australasian Trained Nurses' Association of Sydney. At the present time there are two European probationers. The *Western Pacific Herald* of December 27th, in an article congratulating the authorities of the hospital on the result of the departure originated by the medical department on the appointment of Miss Webster-Wedderburn as matron, has the following:—

> We wish to add a word for the consideration of our mothers and daughters, and to draw their attention seriously to the opening afforded by the nursing profession, not merely for securing an addition to their income, but as a useful and honourable enterprise and benefit to their fellow women and men. The example set by Queen Alexandra, President of that most laudable institution, the Royal National Pension Fund for Nurses, and by the Princess Christian, President of the Royal British Nurses' Association, affords instances not of mere nominal patronage but of a genuine and hard-working devotion to the cause of the nursing profession and of suffering humanity; and the encouraging effects of the magnanimity of those great ladies have already been practically felt even out here in Fiji. Our recruiting field is undoubtedly small in this colony, but past efforts have met with signal success, and there is no reason why we should not look forward to the future with confidence.

### PERSONALITIES AT MALTON.

At a meeting of the Malton Board of Guardians one of the Guardians objected to the continuation of extra diet to the superintendent nurse, who had been suffering from anæmia. He said that the Guardians ought not to be satisfied with the report of the medical officer, but had a right to look into any matter that caused extra expense to the ratepayers. In reply to a question asked by Lady Carlisle, it appeared that the extra diet, which had been continued for six weeks, consisted of a bottle of burgundy at 2s. 2d. and 14 eggs a week instead of three. The objecting Guardian then went on to say that "he did not see why they should pamper servants simply to keep them. The nurse was paid £35 per annum, a higher salary than the matron, and she ought to maintain herself. If she was unable to attend to her duty she must retire, the same as any other officer. She was a favourite with the doctor—was dining with him last Sunday night, and what could they expect the doctor to do? He was not insinuating anything." Possibly not; but we are not surprised that a correspondent, whose letter appears in another column, is indignant at the remarks we have quoted. For a Guardian to speak of a superintendent nurse as a "pampered servant" is neither polite nor politic. It stamps the Board at which such remarks are made, and is sure to add to their difficulties in obtaining nurses.

### A VICTIM OF A LAX SYSTEM.

The death of a "nurse" of twenty-one from typhoid is reported at Little Bromwich Isolation Hospital, and a Birmingham paper commenting on the fact observes that "nurses engaged in nursing typhoid cases run serious risks." Very young nurses are peculiarly susceptible, and there is too much reason to fear that the nursing staff at many of the isolation hospitals is made up with as little regard to physical suitability as to previous training.

### RETIREMENT OF THE CHAIRMAN OF DEAL HOSPITAL.

The new departure at Deal, to which we referred last week, has had the immediate result of causing the retirement of the rector of Deal, who has hitherto acted as chairman of the Deal and Walmer Dispensary and Victoria Cottage Hospital. Mr. Griffiths states that he entirely disapproves of the policy of expansion, and believes that the establishment of a nurses' institute is both inconsistent with the purpose for which the hospital exists, and calculated to impair its efficiency in the future. Of course he does not question the right of the majority of the subscribers to decide the matter, but his withdrawal from a post which he had occupied from the outset is not a happy augury for the success of the policy he condemned.

### SHUTTING THE DOOR ON ROMAN CATHOLIC NURSES.

The Roman Catholic members of the West Derby Board of Guardians were justified in the protest they made at a meeting of the board in Liverpool against the course pursued by the committee with whom the responsibility of appointing the nurses rests. Of five probationers who attended before the committee one only was asked what her religion was, and it seems clear that the inquiry was put in her case because one of her testimonials was from a Roman Catholic priest. She was not selected, and it was stated by several Roman Catholic members of the board that "time after time" candidates had been rejected because of their creed. This should not be; and we agree with the speaker who maintained that there ought to be an end of the policy of exclusion. It is true that some obscure Irish boards of guardians pursue it and shut out Protestant nurses. But guardians in a city like Liverpool should be above such unworthy tactics.

### ASPHYXIATION OF A SISTER AT A BATH HOSPITAL.

The danger of taking soporifics for sleeplessness is illustrated in a striking manner by the sad case of Miss Catherine Thomas, sister in charge of the children's ward at the Royal United Hospital, Bath. Miss Thomas, in spite of being warned by the medical man to whom she was engaged to be married, persisted in using chloroform for insomnia and neuralgia, with the result that a few days ago she was found dead in her bed. There was not the slightest reason for self destruction. Miss Thomas had been at the Royal United Hospital for nine years, and was much esteemed by the matron and the staff; her relations with her fiancé were satisfactory, and her general health was good. As the coroner also said, the medical evidence clearly pointed to an accident. "It seemed probable that the deceased used chloroform to obtain sleep, and then accidentally turned her face on the pillow in such a way that she was asphyxiated." The brother of the unfortunate nurse asked the matron, who deposed that she had not known Miss Thomas to take drugs to induce sleep, "whether it was not a custom amongst some nurses to use chloroform for sleeplessness;" but the coroner ruled the question out of order. Whether it be a custom or not, we hope that the shocking consequences in this instance will act as a deterrent.

### THE WAR NURSES.

The *Britannic* and the *Gaika* have arrived at Southampton. On board the former were H. Snart, A.N.S.R., who requires one month's leave, and returns to South Africa; C. Condell, A.N.S.R., who requires two months' leave, and returns to South Africa; F. L. Carey, A.N.S.R., who requires six weeks' leave, and may possibly return to South Africa; and M. Paulett-Williams, Civil Nurse, who requires three months' leave, and returns to South Africa. On the *Gaika* were two Civil Nurses, viz., B. C. Denmyer and M. Denmyer.

### RESIGNATION OF A HOSPITAL MATRON.

Miss L. N. Rae, lady superintendent and matron for five years of the Coventry and Warwickshire Hospital, has resigned her position. The resignation was accepted at a meeting of the committee last week, after a long discussion, in the course of which it was stated that the step taken by Miss Rae was the sequel of lady visitors having come to the hospital at an inconvenient time and having made complaints. This statement was confirmed by Miss Rae herself, who also expressed the opinion that the House Committee had not backed her up as they might have done. Several speakers referred in complimentary terms to the manner in which the lady superintendent had discharged her duties.

### SHORT ITEMS.

Alderman Duckworth, formerly M.P. for the Middleton division of Lancashire, has contributed £1,000 to the Nurses' Home, which is to be established at Rochdale to commemorate the reign of Queen Victoria.—The sessional lecture to the members of the Royal British Nurses' Association will be given on Tuesday next, at 10 Orchard Street, by Mr. G. D. Leslie, R.A., on "Personal Reminiscences of a Royal Academician," at 5.30 p.m.

### Lectures to Nurses on Anatomy.

By W. Johnson Smith, F.R.C.S., Principal Medical Officer, Seamen's Hospital, Greenwich.

### LECTURE XIII.—STRUCTURE AND DEVELOPMENT OF BONE.

Having so far studied our osteology on dry specimens and the articulated skeleton, we should now endeavour to learn in one or two quite fresh specimens the natural condition, and so far as can be made out by the naked eye, the structure of bones. For this purpose detached bones of a recently killed mammal will suffice.

It will be seen in the first place that in a fresh bone the special features, and even the general outlines are more or less obscured by the attached portions of muscles, tendons, and ligaments. It is very difficult to separate a bone from the surrounding soft parts and to obtain a completely isolated specimen.

In those parts which are not thus obscured we find that our fresh specimen does not present the smooth and hard surface of the museum bones we have hitherto studied. It is covered by a thin, continuous membrane which, especially in the bone of a young animal, can, with the aid of a sharp scalpel and forceps, be readily detached. This membrane, which forms as it were a skin for the bone is the *periosteum* (fig. 31 P). Though thin and not conspicuous, this is a most important structure as it takes part in the growth of the bone, and also in its partial regeneration after the ravages of injury and disease.

Where two or more bones come together to form a joint

Fig. 31.—Section of femur.

the opposed surfaces are covered by a very smooth, firm, elastic structure of a bluish-white colour. The best specimens of this structure, which is called *articular cartilage* (fig. 31 C), will be found on the upper extremities or heads of the thigh and arm bones, and in the portions of bone forming the knee-joint.

In a recent, though not so well as in an old and dry long bone, the surface, especially near the extremities, will be seen perforated here and there by numerous small holes which serve for the passage of blood vessels—growth and vitality in bone as in softer and more active structures depending on a constant renewal of healthy blood.

If a bone be sawn through in different directions it will be seen to be composed of an external layer of hard and dense osseous structure—*compact tissue* (figs. 31, 32 A) and an internal mass of lighter and open or spongy tissue (B) which can be readily crushed. The latter is called *cancellous tissue*, the meshes of which are occupied by a yellowish semi-fluid material like oil or soft fat, called the *marrow or medulla.* In large and long bones, such as those of the limbs, the middle of the shaft is hollow, and this hollow cavity called the *medullary cavity* (figs. 31, 32 C) is lined by an inner skin thinner than the periosteum, which is termed the *medullary membrane.*

*Chemical Composition of Bone.*—A rough and ready analysis suffices to prove that bone is composed partly of animal, partly of earthy matter. If a long bone be kept for about a week in a strong solution of hydrochloric acid it will lose its earthy ingredients, and, whilst preserving its shape,

Fig. 32.—Transverse section of a bone.

be found so flexible that it may without much difficulty be twisted into a knot. On the other hand, by boiling or calcining the bone, the animal matter may be removed, and the bone reduced to a dry and crumbling mass of inorganic material. The animal material, which consists chiefly of gelatine, and renders bone tissue tough and elastic, forms about one-third of a bone; and the earthy matter made up to a great extent of phosphate of lime, bone-earth as it is called, and giving hardness and resistance, forms about two-thirds. This proportion varies in different animals and in the human being at different stages of life. The hard and white bones of birds contain a very large proportion of earthy matter whilst the soft and flexible bones of fishes are composed to a great extent of gelatine. In the elastic bones of the child there is less earthy and more animal matter, whilst in subjects of advanced age the bones are rendered unnaturally brittle in consequence of an increased proportion of the former.

*Development and Growth of Bone.*—The bones of the trunk and limbs in the fœtus or unborn child are composed almost wholly of an animal material known as cartilage. This mass of cartilage, which at a very early period takes the form of the future bone, becomes gradually ossified or converted into hard osseous tissue. This conversion of cartilage into osseous tissue does not affect the whole of the young and growing bone at the same time, but starts

Fig. 33.—Tibia, showing centres of ossification; A, diaphysis or shaft; B, upper and (C) lower epiphyses.

from one or more centres called *centres* or *nuclei of ossification.* For instance, the cartilaginous model of the future tibia begins to ossify in the middle of the shaft about seven months before birth. Soon after birth a second centre of ossification shows itself in the upper extremity of the bone, and in the course of the second year of life a third centre appears near the lower extremity. Ossification spreads gradually from each of these centres until at, or soon after, the age of puberty the bone becomes a single and continuous mass. Until this takes place, however, there are three portions of tibia, an ossifying shaft called the *diaphysis*, and two ossifying extremities called *epiphsyes*, each of which, though completely converted into bone, is, until the eighteenth or twenty-first years of life attached to the rest of the tibia by a thin line of still unossified cartilage termed the *epiphysial line* (fig. 33). Development by a primary and by secondary centres of ossification occurs in most of the bones of the trunk and limbs, but in many of the long bones is more complex. The humerus, for instance, is built up from no less than seven centres, and the femur from five centres.

A knowledge of the development of the different bones helps the expert in forensic medicine in making out approximately the age of a dead infant and the period of life reached by the subject of some unknown human remains. As the growth of a long bone takes place chiefly at the lines separating the shaft from the still separate ossifying centres —the *diaphysis* from the *epiphysis*, removal of this district in operating on the bone for disease would result in arrest of growth and shortening of the limb. Thus, in excision of the knee in a child the surgeon endeavours to saw through the end of the femur below the line separating the ossifying shaft from the ossifying cartilages.

In the flat and expanded bones of the skull-cap ossification takes place from distinct centres, but the model of the future bone is composed not of thick and tough cartilage but of thin and pliant membrane. Thus the head of an infant is soft and yielding and during difficult parturition may be much elongated without any very serious result.

Until the ossification and union of the bones of the cranial vault has been completed certain soft and yielding spots may be felt in the infant's head, especially at the extremities of the sagittal sutures. These yielding parts are called *fontanelles*.

Bones increase in thickness by a deposit of new osseous tissue beneath the periosteum, and in length by a similar deposit to the ends of the shaft near the epiphysial lines. When diaphysis and epiphyses have joined together to form a single and compact bone, growth has, in most long bones, reached its limit.

### The Nursing Question at the Central Poor Law Conference.

### MR. HUMPHREYS' VIEWS.

The thirtieth annual Central Poor Law Conference was held last week in London at the Council Chamber of the Guildhall, Lord Cross presiding. There was an interesting discussion on the nursing difficulty, which followed the reading of a paper by Mr. F. R. Humphreys, L.R.C.P., who started by insisting upon the necessity of making the nursing treatment of the sick poor in workhouse sick wards more efficient. He then proceeded to describe the existing situation. "The sick wards of a workhouse were lacking in everything that made for success or that facilitated the work. The buildings were mostly small and badly built. The essentials of nursing were often wanting, and the medical staff was represented by a single underpaid, overworked official, who lived outside and often at some distance from the infirmary. The nursing staff, even in the better infirmaries, was often made up of one or two well-trained nurses, whose whole time was occupied in filling up forms and writing reports, and who were simply lost in a multitude of 'assistant nurses who were totally untrained.'"

In order to meet these and other objections raised to the present system, and to provide, in an adequate manner, in proper buildings, and by skilled training, for the sick, Mr. Humphreys urged that the disappearance of the small workhouse infirmary was a necessity. How was this, he asked, to be brought about? There were only between 30 and 40 provincial workhouses large enough to properly train probationers, and these could only turn out 60 fully trained nurses in the year. As one nurse to 15 patients was required, there would be 4,200 nurses needed to nurse the 64,000 patients which on an average were to be found in the workhouse infirmaries. He recommended the removal of the sick poor from the smaller infirmaries to large centrally situated institutions, conveniently located for their reception, properly constructed, adequately staffed, and capable of self-support in the training of probationers. These institutions he would call district hospitals. He was convinced that the 652 infirmaries could be reduced to about 148, 92 of which would contain 200 beds or more. Patients should be not further from a district hospital than would entail a journey of 15 miles by road or 30 miles by train. These hospitals should have from 200 to 500 beds. The Welsh infirmaries were in a hopeless condition and something special would have to be done for them.

### Condemnation of the Scheme.

A discussion followed, in the course of which Mr. Tillotson, of Halifax, said that the scheme proposed by Mr. Humphreys was about as impracticable as anything he had ever heard of. He complained very much of the dearth of nurses, declared that their quality was below what it used to be, and agreed that every effort should be made to obtain better ones by offering good salaries and better treatment.

Mr. Vulliamy, of Ipswich, also described the scheme as impracticable. He affirmed that the great need of the guardians was nurses, and expressed the opinion that one reason why they did not get them was that they made too many demands upon them. He thought that the life of the nurse in a workhouse infirmary was too dull, and without the amusement and change which were necessary to render life happy and endurable.

### The Nurses Good Enough.

Dr. J. M. Rhodes, of Charlton, pointed out that during the last six years there had been an enormous improvement in the nursing in workhouse infirmaries. He thought they were going on the right course, and that it was not necessary to adopt any heroic measures to provide nurses. The unfortunate war had taken many nurses from the large workhouse infirmaries. These nurses had been found good enough to nurse His Majesty's soldiers, and surely they were good enough to nurse His Majesty's paupers. No doubt the small boards of guardians had difficulties to contend with, and some of them failed in their duty as regarded their infirmaries. Every effort should be made to bring up these backward boards into line with the others. With regard to Mr. Humphreys' scheme, he need only refer to the fact that each district would cover 250,000 acres, to show that it was self-condemned. What guardian or medical man would like to move, say, a pneumonia patient a distance of 15 miles on a cold winter's night?

Mr. W. Mallalieu, of Shardlow, suggested that proper nurses could be got if the guardians sent young women to study at the large hospitals, and paid their fees for them; and he was followed by Mr. Summers, of Sunderland, who told the Conference that in Sunderland the guardians were doing their "level best" to secure the most efficient attention for the sick poor. The new hospital they were erecting would contain 200 beds and cost £40,000.

The proceedings terminated with a vote of thanks to the President, and to Mr. Humphreys for his paper.

### Six Months' Private Work.

### A NURSE'S PERSONAL EXPERIENCES.

(*Continued from page* 308.)

### A Typhoid Patient.

Here I remained for two days, and on the afternoon of the third I was despatched to my second case. I had some distance to go, and on arrival was met by a young man who turned out to be the patient's brother. The house was small, extremely dirty, and looked as though its occupants had just moved in, and had little or no furniture. The patient and her brother were the sole inmates, but they explained that the patient's husband had gone to the City. I found that my patient was suffering from typhoid, though I had been merely told that Mrs. O. was being treated for hysteria. She was in a most terrible plight, and looked as though nothing had been done for her for a week, as indeed it had not. Her face had not been washed, nor her hair combed for that period, whilst the bed clothing was simply black. The poor creature was really too ill to be questioned, so I held a council of war with the brother as to where I should find clean things, bedding, etc. To my surprise he told me there was no change of sheets in the house, but that he would go out and buy some. He appeared to be anxious to do anything to help me, and indeed in the weary days that followed I know not what I should have done without him. He stated that their present abode was only temporary whilst seeking a fresh business. I set to work with a will, made the bed up with the new sheets, and did my utmost to put the room a little tidy. I had barely finished before the husband of the patient came home and appeared to be glad to find me there. He was very polite, but his style of dress reminded me of the type of sporting men one sees on the stage. He invariably wore a long fawn racing coat, a soft felt hat, and always carried a small leather valise over his arm. During the evening the doctor called, and he gave it as his opinion there was something mysterious about the household. He was most attentive to his patient, but she grew worse every day. I had managed to get a woman to come in in the mornings and clear up a bit, but she refused to do anything in the sick-room, so I had all my work cut out for me. The husband had business which took him to the City every day, whilst the brother used to depart on shopping expeditions, leaving me alone with the patient several hours at a stretch. On the sixth morning every symptom of typhoid had developed. Next day a specialist from the "London" came to see my patient, and a nurse was sent for for night duty, and as there was no sleeping accommodation we arranged to go to and fro to the Home. To make a long story short, all our efforts were in vain, and on the fifteenth day from my first going to her, my patient died. The husband's grief was extreme; he had never seemed to realise that the illness might end fatally, and the saddest part of the story has yet to come. Two days after I had occasion to see the doctor and he told me that the so-called Mrs. O. was not entitled to the name, for although she had been living with Mr. O. for several months he had a wife and children at Newmarket. This explained many things I had thought so strange, and several days later I received a letter from Mr. O., thanking me for my "very kind attention to his late dear love" together with a card inscribed "In loving memory of C., aged 28." About a month after I was very much surprised and shocked to hear of the death from typhoid of the doctor also.

### Scarlet Fever at a Shop.

My third experience was quite a change; four little children with scarlet fever. This was at a shop, and a large room above it, called by courtesy the drawing-room, was given over for the sick room. It is fortunate it was large and airy, for the patients, a small nursemaid and myself, had to live and sleep in it all together. The mother was away from home, having only just recovered from a severe illness, and the father was one of the most uncouth and disagreeable men it has ever been my misfortune to meet. My little patients were not very ill, they had the fever very lightly, and as they were all thoroughly spoilt and used to having their own way, my task was not an easy one. For the first few days I was driven nearly to distraction, for they had a dreadful way of going into a fit of screaming if they were opposed in the slightest degree, but when they got more used to me things went rather more smoothly. However, I had not a nice time by any means, and I could not even have the use of a separate room in which to wash and dress. I managed to secure a washing basin, however, which I had to keep under my bed, and that is all I had to use for six long weeks. I had no place in which to put away my bonnet, and so it usually remained on the top of the piano, for, as I said, we were in the drawing-room. One day, when my back was turned for a few minutes, the youngest child got dragging it round the room by its veil, calling it a cart for his dolly. The six weeks of my stay there seemed interminable, but they came to an end at last, and I was truly thankful to depart, not without much grumbling on the part of the amiable shopkeeper at the fees, and also at the quarantine money; a word of thanks for myself being quite out of the question.

### A Case of Influenza.

After my week's rest, I was dispatched in a great hurry late one night to a gentleman with influenza. This was the nicest case I had had so far, and I appreciated it, although it was very hard work for the first fortnight, as the patient was very ill, and I had very little rest. They were wealthy people, and the house was a very good one, so after my previous cases I felt quite in the lap of luxury. My patient made an excellent recovery, and was able on Christmas day to join us at dinner, to which a small party of friends had been invited. Had I been a daughter of the house, I could not have received more kindness from my patient and his wife, so I began to think that private work had its bright side after all, and the five weeks I remained there passed all too rapidly.

### Night Nursing.

Thence I was sent straight away to one of the London throat hospitals, to do temporary night sister's duty, but I was only required for a few days. Next came a rest of two days in the Home, and then a case near by where I was wanted at night, and had to sleep in the Home, the patient's friends undertaking to look after her during the day, which I found a very unsatisfactory proceeding. My patient was an old lady of 90, who had bronchitis badly, as well as cardiac weakness, but her friends did not seem to realise that she was in any danger, and in spite of all the doctor's orders would insist on knowing what was good for her. There were two daughters and a daughter-in-law, and between them they undid all the good that was ever done for the poor old lady. Just as they were beginning to wake up to the fact that a day nurse was required she died.

(*To be continued.*)

### Nursing at a Small Military Station Hospital.

BY AN ARMY RESERVE SISTER.

ABOUT two years ago, burning with enthusiasm, and suffering from a bad attack of war fever, I joined the Army Nursing Service Reserve. Of course my aim and object was South Africa, but I also had a dim and hazy notion of putting things straight in China. However, the War Office did not seem to realise what a bright and shining light had joined their ranks, and I was sent to a small station hospital in England, and there I have been ever since. I got my orders for South Africa duly, but could not persuade the too conscientious medical officer to pass me as medically fit, so I had to content myself, with many sighs, to an easy life in the station hospital.

### A CONTRAST TO A CIVIL HOSPITAL.

I wonder if all nurses, straight from training, feel so utterly at a loss on first entering a military hospital as I did. Everything is so totally different to what one is accustomed to. The wards here are large, 25 beds in each; they are very bare and like a barrack-room. The floors and lockers are plain scrubbed wood. Everything looks remarkably clean and neat in the morning, but as the majority of the patients are able to be up and about, it is rather difficult to make them realise the importance of tidiness throughout the rest of the day. Nowadays one hears much about reform in the medical and nursing services in the Army, and people who really know very little about it, write at great length, and air their opinions on the subject. But I think very few realise for what very trivial ailments poor "Tommy" has to "go sick." It is not his own fault, as a rule; unless he is a confirmed loafer he does not care for a rest in hospital, as part of his pay is stopped during his sojourn there. Yet he must either be in robust health and able to do all his duty, or else he must be in hospital—there are no half measures for him.

### LIFE EASY FOR THE SISTER.

Of course one never gets a very old man as a patient in a military hospital; the majority of men are quite young, and as they all have to pass a stringent medical examination before being passed into the army, they all have fairly good constitutions. So, though the number of patients would make one very busy in a civil hospital, yet here the majority of cases are so mild that life is very easy for the sister. We have a ward set apart for the special and acute cases, and sometimes in that ward there is really a great deal to do. But on an average there will not be more than four or five acute cases in the entire hospital, and those mostly medical. The surgical work is very slight. Sometimes, of course, a small operation is done; but as a rule, all the important operation cases are sent to Netley. One may have a surgical ward of 25 beds, and each man will have a small dressing to be done; but slight cuts, bruises, sprained ankles, gum boils, etc., form the majority of the complaints. Accordingly, in that ward, most of the patients are able to wash themselves, make their own beds, and even scrub their own lockers and part of the ward floor.

### ORDERLIES AND WARD MASTER.

We have all civilian or regimental orderlies, nearly all the R.A.M.C. being out in South Africa. The sisters take all the temperatures, give all medicines, and do all the dressing. Also, for the acute cases, they make the beds and do all the sponging, feeding, etc. The orderlies are responsible for the cleanliness and equipment of the wards, also for all the diets. There is a very grand person, called a ward master, who is generally a sergeant, and who is supposed to superintend the orderlies in two or three wards and to see that proper military discipline is maintained; he also appoints convalescent patients for fatigue duties, as they are called, such as digging in the garden, cleaning windows, and so on. Then we have a sergeant-major, who is really wonderful; he is the most diplomatic man I ever met. Everyone goes to him with all their difficulties. He helps them all or pacifies them, but never in any quarrel sides with one party more than another.

### SPLENDID PATIENTS.

Taking them all round, the soldiers make splendid patients; they hate to be fussed over, and like one to do just what is necessary for them and then depart. One has to use a good deal of tact sometimes to make things work smoothly, but generally the orderlies and patients will do anything for the sister if she is ordinarily nice to them. It is a fatal mistake to report either orderly or patient unless absolutely obliged. They will forgive any amount of personal scolding, and sometimes we have to "go" for them very vigorously, but if a sister begins reporting unnecessarily, her life is made a burden to her.

### HOURS OF DUTY.

In this hospital the sisters have each a little room to sit in; it is called the "Bunk," for some unknown reason, and is at the end of the ward; there is a window in it from which we can completely overlook the ward. All poultices, fomentations, etc., are made in this little room, and all the patients who are able to walk come to it to be dressed. The sisters go on duty at 9 A.M. At 1 P.M. they have lunch, at 2 return to the wards, give medicines, and do everything there is to do, and then are off duty till 6 P.M., then again on duty till 9 P.M. They take it in turns to be on orderly duty; their day on duty they are responsible for the entire hospital from 2.30 till 6 P.M. Sometimes we have one night sister, sometimes two; it depends on the work. They are on duty from 9 P.M. till 9 A.M. As a rule the night duty is not very trying. I never met patients who sleep so well as the soldiers do, they have to be very ill indeed to be at all wakeful. Each medical officer has his own wards, and visits between 10 and 11 A.M.; but in the evening there is an orderly M.O. who visits all the wards, though if any M.O. has an acute case in his ward he is supposed to visit it himself in the evening, the orderly M.O. being principally for the newly admitted and detained cases. On first going to a military hospital one feels rather hopeless, it seems so difficult to get things for use, but after I had been here for a while I began to understand the workings of things, and now I find I can get pretty nearly all I want, in reason, if only I set about it in the right way. Of course these remarks only apply to the small hospital where I have been stationed. I have no personal knowledge of the workings of a larger institution of the same kind.

### To Nurses.

**WE invite contributions from any of our readers, and shall be glad to pay for "Notes on News from the Nursing World," or for articles describing nursing experiences, or dealing with any nursing question from an original point of view. The minimum payment for contributions is 5s., but we welcome interesting contributions of a column, or a page, in length. It may be added that notices of appointments, entertainments, presentations, and deaths are not paid for, but that we are always glad to receive them. All rejected manuscripts are returned in due course, and all payments for manuscripts used are made as early as possible after the beginning of each quarter.**

### The Treatment of Bedsores.

### EXAMINATION QUESTIONS FOR NURSES.

The question was as follows:—"How would you treat bedsores in their various stages if the medical man left such treatment to your discretion?"

### First Prize.

*In the First Stage*, where the skin is merely rubbed off, the affected parts may be washed frequently with soap and water and wiped gently with a soft towel. Afterwards put a little zinc ointment on with the finger, or apply powder—starch, zinc and boracic, equal parts, mixed.

*Second Stage.*—Where the skin is off and a raw surface exposed. Bathe the sore with a stimulating lotion, if patient can bear it; otherwise boracic is good. Spread boracic ointment on lint cut exactly the size of sore, place a piece of lint rather larger over this, to avoid the strapping breaking down the edges, fastening on with strapping. Mercurial ointment is good to stimulate. Red lotion may be tried instead of boracic ointment, but seldom zinc ointment, because it cakes. Dress this sore only once or twice daily.

*Third Stage*—A sloughing sore. Bathe, or better still, syringe gently with weak antiseptic lotion. Apply frequently boracic fomentations till the slough disappears; then either heal with fomentations, or a healing ointment can be used. If the sore is large, use a bandage instead of strapping.

*Fourth Stage.*—A gangrenous sore (seldom left entirely to a nurse). Linseed and charcoal poultices are good, clearing away the dead flesh and putrid matter. Before each poultice the sore should be clean-ed, and often dead pieces may be cut away with the help of scissors and forceps. When the sore is clear a deep cavity generally remains. This should be plugged with gauze or lint and dressed as an ordinary wound. It should heal up from the bottom.

*Fifth Stage*—Where the sore is alive, generally through lying for days on a wet feather bed. Apply fomentation of perchloride of mercury. The patient rarely, if ever, recovers. In the general treatment of all stages the bed must be kept clean, dry, and unwrinkled. Even in the poorest houses, if a nurse attends, it is possible to get a mackintosh, water-pillow, and clean linen. If the patient lies on a feather bed, remove if possible, Otherwise sink the water-pillow right into the bed, quite flat. Place pads or pillows between the knees and ankles. A pad of wool or tow should never be placed under the back. Should the patient have incontinence it would make a poultice and sodden the skin; any way it is irritating and heating. A ring of wool linen covered, can be placed under sore heels to prevent them touching the bed. The patient should be kept off the sores if possible, and frequently the position in bed changed. Sometimes a patient is unable to keep on one side when turned, but falls back into his old position. This may be often avoided by pillows placed against the back.

Villa.

### Second Prize.

In all cases and stages of bedsores I should have the back of the patient thoroughly washed with soap and warm water, and thoroughly dried with a fine cloth. In some cases I would rub the skin with methylated spirit or whisky until dry (avoiding the sores). In other cases, where there may be much moisture, either from perspiration or passing urine unconsciously, I should rub the back with oil, vaseline, or zinc ointment until the skin has absorbed the lubricant. This will preserve the surrounding tissues, also help circulation and aid healing to the dead tissues. Also have the pressure removed from hard mattresses by placing a water-pillow under the patient's back, but in extreme cases a water-bed ought to be procured.

*A Surface Bedsore* may be healed with care, and washing the skin with soap and water, dried, dusted with a little zinc or starch powder, dress the sores with zinc, vaseline, or boracic ointments.

*A Neglected Bedsore*, where the inflammation may have got into the surrounding tissues of the back and formed a quantity of pus. In this case I should be directed by the medical attendant, and probably an antiseptic may be ordered, such as Condy's fluid, carbolic lotion 1-40. If so, syringing at this stage may prove beneficial to clean thoroughly. I should endeavour to heal all bedsores from the foundation. A dry dressing may be used at this stage, such as iodoform gauze or boracic lint and powder.

*A Sloughing Bedsore.*—A moist dressing of boracic lint, or lint wrung out of a weak carbolic lotion 1-50, and covered with oil-skin, will clean away the dirty matter.

*Another Stage.*—When a bedsore is not making any progress in healing, after trying various remedies, red lotion may be used for a day or two, applied on a piece of surgeon's lint, to stimulate the tissues. I should change all those dressings three times a day, and once during the night. When healing has begun I should resort again to the simpler dressings. I would have the dressings no larger than the sores, and have the patient frequently moved on the most comfortable side. I should always have a smooth draw-sheet under a patient, and always turn a patient from side to side on a draw-sheet. And if a patient is restless a triangular bandage would keep dressing in position. In extreme cases it is better not to attempt to put patient on the bed-pan, as it may retard healing of the sores. Rather have pads made of old linen, which can easily be changed, without much fatigue to patient. Patient's garments ought to be of soft material, also without any creases underneath, either made short or to fasten at back. If evacuations are involuntary, the back ought to be attended to after every motion, or urine passed, to prevent any moisture getting into the sores, as it would prevent the healing process.

Magdalane.

### Are the Answers as Good as they should be?

Not quite, I think. There is too much running in a groove. The treatment of bedsores is one that allows free scope to a nurse's individual ingenuity and powers of observation. Doctors seldom interfere much in the matter for two reasons—first, they feel that such prevention and treatment is peculiarly the nurse's province; and secondly, because they thoroughly understand that constant change of arrangement is necessary.

It is the apparent paucity of ideas as to different remedies of which I complain this month. Boracic acid is an excellent servant, but do not let it become entirely our master. Few nurses seem to be able to rise above or sink below this valuable remedy.

"Villa" is successful as the first-prize winner because she mentions bedsores in all, and every, horrible condition, and also because she has resources. She knows only too well the difficulty of banishing the ancestral feather bed. True, but her constructive genius overcomes the mountain.

### Honourable Mention.

Three very good answers have gained Honourable Mention: "Anin," who was also successful last month, "Nurse M.," and "The Tappa." The last-mentioned nurse in her noble work has probably great opportunities for observing this painful development of illness.

### Question for March.

If in a case of typhoid fever the doctor ordered sponging of the entire body, how would you proceed to carry out his orders?

### Foreign and Colonial Nurses.

Requests having reached me from readers beyond the seas that time should be allowed for them to compete in these examinations, the matter is under consideration, and the decision will, if possible, be announced in April.

The Examiner.

### Rules.

The competition is open to all. Answers must not exceed 500 words, and be written on one side of the paper only. The pseudonym, as well as the proper name and address, must be written on the *same* paper, and *not on a separate sheet*. Papers may be sent in for fifteen days only from the day of the publication of the question. All illustrations strictly prohibited. Failure to comply with these rules will disqualify the candidate for competition. Prizes will be awarded for the two best answers. Papers to be sent to "The Editor," with "Examination" written on the left-hand corner of the envelope.

N.B.—The decision of the examiners is final, and no correspondence on the subject can be entertained.

In addition to two prizes honourable mention cards will be awarded to those who have sent in exceptionally good papers.

### The Nurses' Bookshelf.

SYLLABUS OF LECTURES TO NURSES. By ANDREW DAVIDSON, M.D. (The Scientific Press, Limited, 28 & 29 Southampton Street, Strand, W.C. January 17th, 1902. 100 pages. Price 1s.)

WE cannot help thinking that, if a course of lectures is to reach the highest standard of excellence, the lecturer must himself select the headings under which he divides his subject as well as the details which come under each heading. But a busy medical man may be asked to deliver a course of lectures to nurses under circumstances which render a refusal ungracious, so that he is obliged to undertake the work, although he may have but little time to devote to its preparation. He will not like to buy a complete set of lectures and read them to his class, because any man, selected for such a duty, is certain to have ideas of his own and facts concerning nursing gathered from his own experience, which he would wish to communicate. Now, in a case of this kind, Dr. Davidson's book will be of the greatest service, for it is the arrangement of the headings which takes time, and these are amply supplied in the syllabus. The plan of the book is that on each left-hand page there are printed headings, *e.g.*, *Diarrhœa* (1) number of motions; (2) amount; (3) colour and so on; while the right-hand page is left blank for the lecturer's own notes. The result is that while the lecturer is saved the trouble of arranging his matter, he can nevertheless give, under each heading, his own thoughts and views. It would be quite possible for a well-informed man, with this book in his hand, to give a series of good lectures without any preparation whatever. The subjects included in the syllabus are anatomy, physiology, first aid, and the care of the sick, with a special chapter on the care of the insane. The headings are very numerous, and it must have been a difficult matter to decide what to put in and what to leave out. In some places there seems to us to be too much detail, as when the component parts of the teeth, enamel, dentine, etc., are all mentioned. In other places there are curious omissions; for instance, the ovaries are not included among the organs within the pelvis. The most serious omission which we noticed is that the nose is not mentioned among the respiratory passages, in spite of the importance of its functions and the danger of breathing through the mouth.

ENGLISHWOMAN'S YEAR BOOK AND DIRECTORY. Edited by EMILY JANES. (London: A. and J. Black. 1902. Price 2s. 6d. net.)

THIS very indispensable book of reference has reached its twenty-second year, and under its able editor appears again for the guidance of women who, "professionally or socially are brought in touch with the multifarious needs of modern life." The present issue has been carefully revised and much new information has been added. Each section has had the benefit of expert supervision by ladies whose names are identified with the particular subject. Comprehensive and concise information is given upon every sphere open to women workers. Medicine, science, music, literature, art, etc., while sports, pastimes, and social life are also noticed. The benevolent will find reliable information in the Philanthropy Section. In a broad-minded and sympathetic introduction by the editor, to those women who may use her book, it is evident that her aim is other than the mere tabulation of details. She urges upon them the necessity of work for work's sake and from the highest motives. They must realise the mind of the age and importance of individual life and personal effort. "New opportunities are springing up through the war, for the services of trained and womanly women who shall be helpmeets to the men to whose hands the establishment of a real civilisation has fallen." To this annually-increasing class of women the "Englishwoman's Year Book" will be of invaluable assistance.

### Everybody's Opinion.

**[Correspondence on all subjects is invited, but we cannot in any way be responsible for the opinions expressed by our correspondents. No communication can be entertained if the name and address of the correspondent are not given as a guarantee of good faith, but not necessarily for publication. All correspondents should write on one side of the paper only.]**

### THE CONCENTRATION CAMPS.

"MISS G. L. SHELBY" writes from No. 20 General Hospital Elandsfontein, Transvaal, under date of Feb. 7: Allow me to contradict the statement in your issue of Dec. 28 that I have gone to nurse in the Boer camps. The Miss Shelby appointed must be another lady of the same name.

### DOUGLAS ISOLATION HOSPITAL.

"E. P. PRIEST" writes from the City Hospital, Fazakerley, Liverpool: Seeing that someone has incorrectly inserted the notice of my appointment in last week's HOSPITAL, I shall be glad if you will kindly have it put in again next week correctly as follows:—

Douglas Isolation Hospital, I.O.M.—Miss Eva Priest has been appointed matron of this hospital. She was trained for three years at the Liverpool Workhouse Hospital, Brownlow Hill, and was afterwards district nurse at Gresford, N. Wales, charge nurse at the City Hospital N., Liverpool, charge nurse at the Park Fever Hospital, London, and night superintendent and assistant matron at the City Hospital, Fazakerley, Liverpool.

[The appointment was inserted as sent from official sources.—ED. HOSPITAL.]

### A BRAVE YOUNG NURSE.

"THE REV. F——" writes: The other afternoon a poor little ragged urchin, 10 or 11 years of age, with bare feet, and holding a bundle of matches in his grimy hands, attempted to cross one of the most crowded thoroughfares in Croydon; just as he reached the centre of the roadway, a van, drawn by two spirited horses dashed up—a shriek, a cry, then like a flash a bright young nurse sprang forward, with one hand seizing the boy by his ragged coat, and with the other the bridles of the horses, she managed, unaided, to fling the lad out of harm's way. It was but the work of a moment; yet one shudders to think what might have happened to that young and useful life, so regardless of self, but whose one aim is "to help the helpless," come what may. Amongst all who witnessed the incident, no one seemed to notice the piteous whines and cries of a small brown dog, and the delight with which the faithful little creature greeted his brave young mistress's safe return to his side.

### NURSING IN WORKHOUSE INFIRMARIES.

"A LOVER OF JUSTICE AND TRUTH" writes: You and your readers will probably have seen a report which appeared in the local paper of March 1st of a meeting of the Board of Guardians, at which one of the guardians made some most uncalled for references to the nurse who has occupied the post of superintendent nurse at Malton Infirmary for over two years. I believe an apology has been made, but surely the matter should be the subject of investigation by the Local Government Board, in order that workhouse nurses may in the future be protected from the type of guardian who, I am afraid, is well known to exist. Nurses will continue to fight shy of workhouse posts while this state of things goes on, and it is not surprising, for workhouse nursing is hard enough, and in country towns such as Malton most unsatisfactory, without the risk of such unwarrantable insinuations. The superintendent nurse ts a conscientious and hard worker, and has laboured under great difficulties most of the time, for Malton Union is not by any means a model or a modern union. I should be glad to know from your readers if it is unusual in union infirmaries to supply the staff with extra diet, etc., if considered necessary by the medical officer.

### Appointments.

[No charge is made for announcements under this head, and we are always glad to receive, and publish, appointments. But it is essential that in all cases the school of training should be given.]

Blackburn Infirmary.—Miss Rosa C. B. Watts has been appointed night superintendent. She was trained for three years at the Poplar and Stepney Sick Asylum, where she has since filled the posts of staff nurse 12 months, ward sister 14 months, and theatre sister 13 months.

Coton Hill Lunatic Asylum, Stafford.—Miss Jane Blair Craig has been appointed matron. She was trained at the Western Infirmary, Glasgow, was for eight years sister in the Victoria Infirmary, Glasgow, and at the time of her appointment to Coton Hill Asylum had been for nine months one of the assistant matrons at the Stirling District Asylum, Larbert, N.B.

Gravesend Hospital.—Miss L. Mary Paine has been appointed matron. She was trained at the Queen's Hospital, Birmingham, for three years. She has since been night superintendent at the Walsall and District Hospital, taking matron's holiday duty for three months; and ward sister and assistant matron at the Seamen's Hospital, Greenwich.

Hull Infirmary.—Miss Lucy Binns has been appointed lady superintendent. She was trained for three years at Leeds General Infirmary, where she was afterwards theatre sister for a year. She has since been night superintendent and assistant matron at Derby Royal Infirmary.

Lambeth Union Children's Infirmary and Isolation Block.—Miss Elizabeth White has been appointed superintendent nurse. She was trained at St. George's-in-the-East Infirmary for three years. She was afterwards ward nurse in the same institution, and has since been superintendent nurse at the Central London District Schools, Hanwell.

Langley Park Infectious Hospital, Lanchester.—Miss Nancy Austin has been appointed nurse. She was trained at St. Mary's Hospital, Paddington, and has since done two and a half years' private nursing, been three years nurse at Dover Fever Hospital, five years at Worcester Union Infirmary, and one year at Chertsey Isolation Hospital.

Maidenhead Workhouse Infirmary.—Miss Amy Laird has been appointed assistant nurse. She was trained by the Meath Workhouse Nursing Association at St. Peter's Home, Maybury Hill, Woking.

Oxford Eye Hospital.—Miss Wilhelmina L. E. Ellis has been appointed charge nurse. She was trained at Leicester Infirmary.

Rawcliffe Hospital and Chorley Dispensary, Chorley.—Miss Janet Little Burgess has been appointed sister-in-charge. She was trained at Carlow County Infirmary for three years, and has since been charge nurse at the Samaritan Free Hospital, London; night sister at the East Suffolk and Ipswich Hospital; and senior sister at the Chelsea Hospital for Women.

Royal Hospital for Incurables, Donnybrook, Dublin.—Miss M. C. Beavan has been appointed night superintendent. She was trained, for one year, at the General Hospital, Cheltenham, and now holds the certificate of the Adelaide Hospital, Dublin, to which institution she was attached for five years.

St. Mary's Infirmary, Islington.—Miss J. Parker and Miss Edith Ramsey have been appointed sisters. Miss Parker was trained at the County Infirmary, Londonderry. Miss Ramsey was trained at Guy's Hospital, London, and has since been night superintendent at the General Hospital Tunbridge Wells.

Swansea Union Workhouse.—Miss Mary Tayler has been appointed charge nurse. She was trained at Sheffield Union Infirmary.

Ulverston Sanatorium.—Miss E. Watson has been appointed nurse-matron. She was trained at Salford Union Infirmary, and has since been superintendent nurse a Lanchester Union Infirmary.

Walsall and District Hospital.—Miss Florence Davis has been appointed matron. She was trained for three years at the Guest Hospital, Dudley, where she was afterwards staff nurse and sister. Since July 1899 she has been matron at the Cottage Hospital, Whitchurch, Salop.

Wolverhampton General Hospital.—Miss F. E. Gibbon has been appointed night superintendent. She was trained at the Liverpool Royal Infirmary, and has since held the post of theatre sister at the Cumberland Infirmary, Carlisle, for two years.

### Death in Our Ranks.

We are asked to announce the death of Miss Kate Jung, matron of the Refugee Camp at Brandfort, South Africa, which occurred last October. Her friends in England, however, do not seem to have heard of the sad event at the time it occurred. Miss Jung was a member of the Nurses' Co-operation, New Cavendish Street, and early in 1901 took a voyage to South Africa in order to recruit her health. While staying at Capetown she applied for and obtained the post which she was occupying when she was attacked by dysentery. She died after four days' illness.

### Presentations.

Ealing Parish Nurse.—Miss Warboys, who has been parish nurse at Ealing for upwards of seven years has been presented with a handsome lever clock by numerous subscribers, as a slight testimony of their appreciation of her kind and valuable services.

Prescot Union Infirmary, Whiston.—Miss Jessie H. Grimshaw, on her retirement from the post of Superintendent of Nurses at Prescot Union Infirmary, after nearly three years' work, has received a very handsome silver toilet set, of exquisite design, from the nursing staff as a token of regard.

Portsmouth Workhouse Infirmary.—Miss Ancell, who has recently joined the Portsmouth Parish Infirmary staff as probationer, was, on Sunday afternoon, the recipient of a very useful gift, namely, a well-filled nurse's chatelaine, from the little scholars and fellow teachers of the Sunday School of St. Jude's, Southsea, where she has taught for ten years. In presenting the little gift the vicar, Canon Blake, dwelt upon the perseverance in her work Miss Ancell had shown, and in replying on her behalf, both the medical superintendent and a member of the infirmary committee emphasised the need of bringing such good enduring qualities to a training school, where a high standard was expected.

### Novelties for Nurses.

By Our Shopping Correspondent.

### A USEFUL CLASP.

A clasp for fastening apron and other bands has been patented by Miss Goulding, being the invention of herself and Miss Place. It has been found useful by a nurse who has tried it, and as it is both simple and cheap I may recommend those who find it difficult to keep their belts and straps tidily secured, to write to the inventor for a specimen. It is in the form of two clips, which are kept in position by a toothed edge, and the two sides, when joined, have the appearance of a small buckle. The price is 7½d., and the inventor tells me she hopes soon to be able to sell it at a cheaper rate. Her address is Oletha, Porthcawl, South Wales.

### Nursing in Bombay Hospitals.

HELP FROM GOVERNMENT.

THE following resolution on grants-in-aid of nursing at Government Hospitals has been issued by the Governor of Bombay:—

1. The attention of Government having been drawn to the desirability of improving the existing arrangements for the provision of trained nurses in the large hospitals of the Bombay Presidency, his Excellency the Governor in Council has had under consideration a scheme in accordance with which grants-in-aid of nursing should, subject to the exigencies of the financial situation, be admissible to those Government hospitals in the case of which certain specified conditions may be fulfilled. It seems to the Governor in Council that by providing them with such staff of nurses as may be found requisite, the efficiency of the hospitals would be increased, and at the same time additional facilities would be afforded for the training of a body of qualified local nurses who would be available subsequently to attend to private cases, or for employment in the event of the outbreak of any serious epidemic.

2. In order, therefore, to encourage the provision of adequate nursing establishments in hospitals under the control of Government, the Governor in Council is pleased to direct that any such hospital will be eligible for a special grant-in-aid for this purpose subject to the following rules.—

(1) The grant shall be equal to half the expenditure on the maintenance of the nurses employed at the hospital, provided that the number and emoluments of the nurses so employed shall not exceed the scale prescribed by the Surgeon-General.

(2) The balance of the expenditure on the nursing establishment shall be met from one or any of the following sources:—

(*a*) Endowment Fund.
(*b*) Private subscriptions.
(*c*) Contribution from local body.

(3) A separate account shall be kept of the Nursing Fund of each hospital in such form as may be prescribed by the Surgeon-General, and such account shall be submitted annually to that officer.

The account referred to in Rule 3 should be submitted by those institutions also which are already in receipt of grants-in-aid of nursing.

3. The existing grant-in-aid of nursing to some of the hospitals in Bombay is in excess of half the cost of the establishment maintained. It is not desired to reduce these grants below their present figure, but Government will not increase them unless and until they can do so in accordance with the rules now laid down.

4. The Surgeon-General should be requested to bring these rules to the notice of the authorities concerned in the management of and the supply of nurses to the various hospitals in Bombay and other large towns in the Presidency, and in consultation with such authorities to submit for the consideration of Government the claims which may be put forward in respect of either a new grant, or an addition to one which has already been sanctioned. The claim of each hospital applying under the rules should be submitted separately with a full report as to the circumstances of the institution concerned.

5. The question of the best method of improving the facilities for the training of local nurses is one concerning which Government are not at present in possession of sufficient information to enable them to issue any definitive orders. The Surgeon-General should be requested to obtain, in such way as he may deem fittest, the opinion in this matter of the medical profession in Bombay and submit a full report containing proposals for the consideration of Government.

### For Reading to the Sick.

"BY THY CROSS AND PASSION."

"LORD, when Thy kingdom comes, remember me":
Thus spake the dying lips to dying ears.
O faith, which in that darkest hour could see
The promised glory of the far-off years!

Yet hear the Words the dying Saviour saith:
"Thou too shalt rest in Paradise to-day."
O words of love to answer words of faith!
O words of hope for those who live to pray!

Lord, when with dying lips my prayer is said,
Grant that in faith Thy kingdom I may see,
And, thinking on Thy Cross and bleeding head,
May breathe my parting words, "Remember me."

Remember me, and ere I pass away
Speak Thou th' assuring Word that sets us free,
And make Thy promise to my heart, "To-day
Thou too shalt rest in Paradise with Me."

*Maclagan.*

We have contemplated the Christ, first, as the Great Sympathiser, by reason of His infinite acquaintance with grief. We have seen Him also as the Great Intercessor, by the pleading voice of that sorrow like unto no other sorrow, by His exceeding sorrowful soul offered for sin, poured out to death. Now He stands before us as the Great Life-giver, through that supreme suffering of His. And when we realise all of human sorrow that the union of His Godhead and Manhood involved, we cannot but see Him supreme in sorrow, as supreme in love, in holiness, in beauty; solitary in grief—as some mountain peak, rising far above its fellows in white forlornness, exposed to every blast and all intensity of frost, while sheltering its brethren of less height.

How beautiful, how comforting, to see Him thus! since this is a world of sorrow, and we are men of grief. What confidence it gives us in going to Him with our troubles, to know Him by this character. He is not too far above us to understand and feel for us; He can be touched with the feeling of our infirmities, whatsoever they be. He is not out of our reach, out of touch with us, in the inaccessible recesses of unalloyed happiness. He has perfect human experience, to inform His perfect Divine power of sympathy and aid. Is not such sympathy in itself almost sufficient to heal? And not only in the days of His flesh was He a Man of Sorrows and acquainted with grief. He *is*—still *is*—what He was then. Having taken our nature to the Father's throne, He retains there all His limitless acquaintance with grief. So that, even in the midst of the increased joy that He has won, He may be thus described, we may think of Him as such.—*St. B. Hill.*

"I watched for Thee
On mountains lone all night:
Athirst, for love of Thee, I toiled
All through the hours of light:
In meekness and in lowliness,
In weariness and pain,
I spent my life, I died my death,
Thy dead, lost soul to gain;
And on my heart I bear Thee still
That Thou with me may'st reign."

*Lyra Mystica.*

### Echoes from the Outside World.

### Movements of Royalty.

The first Court of King Edward VII. was held on Friday last. To a certain extent these "Courts" are to take the place of the Victorian Drawing-room, but in two essential particulars they are very different. The Courts take place in the evening, and are by invitation only. The brilliance of the scene at the great State function was heightened by the recent decorations and alterations which Buckingham Palace has undergone, by the installation of electric light, and by the mass of lovely flowers. The King and Queen arrived soon after 10 o'clock, and the guests numbered over 1,000, many of them belonging to the Corps Diplomatique. The reception was in the ball-room. Their Majesties stood on a rug, not on a raised platform. The lady to be presented advanced, and as the Lord Chamberlain—who stood on the left of the King—called out her name, she curtsied and received a gracious bow from the King. A few steps farther on, and a second curtsey was made to the Queen, and then the lady made her way to the door. The Royal Princes and Princesses were grouped behind the monarch, and the ball-room was gay with the official and diplomatic ladies who, unlike those simply presented to their Majesties, are allowed to remain. The guests were supplied with buffet refreshments, and supper was served to the Royal personages. The ceremony terminated shortly before 12 o'clock.

The King is going to give a dinner to half a million of the very poor of London in the week after the Coronation. This announcement was made at the Mansion House on Tuesday by the Lord Mayor, who also stated that his Majesty was prepared to expend the sum of £30,000, which he thinks will be sufficient for the purpose. At the suggestion of the King, the Lord Mayor will undertake the organisation of the entertainment with the co-operation of the borough mayors, of the Chairman of the County Council, and of Sir Thomas Lipton, who was connected with a similar festivity at the celebration of the late Queen's Diamond Jubilee.

The Prince and Princess of Wales celebrated St. Patrick's Day in a practical manner by attending the sale inaugurated by the Irish Industries Association, and held by the permission of Lord Salisbury at his house in Arlington Street. There was a splendid show of work, including much beautiful lace, and the Princess of Wales—who, as well as her husband, wore the shamrock—made a great number of purchases of all sorts besides giving many orders. The keen interest she showed in the productions of the Emerald Isle should go far to increase the popularity of Irish work.

On Wednesday, last week, the Prince and Princess of Wales visited Manchester to inaugurate the new Whitworth Hall at Owen's College. The Lord Mayor of Manchester, the Lady Mayoress, and the Corporation met their distinguished visitors at the station, and a procession of 13 carriages passed through the densely-crowded streets. Admirable order was maintained, and the Prince afterwards wrote to thank the authorities for the manner in which the arrangements had been planned and carried out, saying that he and the Princess hoped the large number of children they were so gratified to notice on the route had reached their homes without accident. The Duke of Devonshire, as president of the College, did the honours, received the party in the quadrangle, and conducted the Prince and Princess to the hall. The Princess wore a heliotrope costume, a sable collarette and muff, and a toque covered with Parma violets. In the course of the Prince of Wales's clear and comprehensive speech, he alluded to the pleasure that it gave him to be present on the fiftieth anniversary of the opening of that College, and said that the work of such an institution must continue to expand, and that it would be a matter of pride and satisfaction to him and the Princess if the outcome of the proceedings was the wiping out of the debt. On the way back to the station the royal procession halted for the unveiling of a statue of the late Queen, the work and gift of Princess Louise, Duchess of Argyll. It stands near the west porch of the Cathedral.

Last week Princess Louise, accompanied by her husband, the Duke of Argyll, visited Liverpool from Knowsley—the seat of the Earl and Countess of Derby—and opened a Navy League bazaar in St. George's Hall. The object of the bazaar is to provide additional funds for the establishment of a Sea Training Home for 250 poor boys at Liscard, on the Cheshire side of the Mersey. Half the purchase-money for the house and grounds of six acres has been already paid, but a balance of £3,000 debt remains. During her visit to Lancashire Princess Louise formally opened the new Northern Hospital. The Lord Mayor of Liverpool, in asking Her Royal Highness to declare the hospital open, presented her with a gold key with a diamond and turquoise head bearing the royal coat of arms, garter and motto. The head of the key can be used as a brooch or pendant. Princess Louise said that she felt it to be a very proud moment that it had fallen to her lot to declare the hospital open. The royal visitor also found time to visit the Walker Art Gallery and inspect the Queen Victoria memorial model before her return to London.

### Foreign.

A decree has been issued by the Empress Dowager of China prohibiting the practice of foot-binding, which has been in operation for very many centuries. An effort to render it illegal was made in the second half of the seventeenth century by the Manchu Emperor Shun Chih, who ordered that parents who bound the feet of their daughters should be put to death. Owing to the opposition of the Board of Rites of the day, however, this edict was repealed only three years after its promulgation, and, except in certain divisions of the Chinese Empire, the custom has prevailed ever since. Whether it has now finally received its death-blow, and Chinese women have been finally delivered from the tyranny of the "lily feet," remains to be seen.

### South Africa.

Swiftly following on the news of Lord Methuen's capture came the intelligence that the wounded general, accompanied by Colonel Townsend, and under care of a medical officer, had been sent into Klerksdorp. On Saturday he arrived at Johannesburg, and the same day Lady Methuen, on her arrival at Southampton from the Cape, had the satisfaction of hearing both of her husband's release and that he was doing well. She, however, proceeded again to the Cape the same afternoon in the *Walmer Castle.*

### Parliament and Politics.

Political, not personal, reasons are the cause of the postponement of the Royal visit to Ireland. Lord Cadogan, in a speech which he delivered in Dublin on Friday, made it very clear that the King had only relinquished the idea with reluctance in consequence of the advice of his Ministers. The Lord Lieutenant expressed the opinion that if his Majesty had visited the sister island during the present year he would have met with a cordial and enthusiastic reception. He, however, accepted his full share in the responsibility of advising the Sovereign to delay the visit until circumstances are more propitious. Meanwhile, intense disappointment has been manifested in almost all parts of Ireland that the people will not have the pleasure—and the advantage—of welcoming the Sovereign and his consort in the year of their Coronation.

### Notes and Queries.

**The Editor is always willing to answer in this column, without any fee, all reasonable questions, as soon as possible.**

**But the following rules must be carefully observed :—**

1. **Every communication must be accompanied by the name and address of the writer.**
2. **The question must always bear upon nursing, directly or indirectly.**

**If an answer is required by letter a fee of half-a-crown must be enclosed with the note containing the inquiry, and we cannot undertake to forward letters addressed to correspondents making inquiries. It is therefore requested that our readers will not enclose either a stamp or a stamped envelope.**

---

*Home.*

(215) Can you tell me of a home where a nurse, convalescent from acute rheumatism, could go for a month or six weeks ?—*H.H.*

The House of Rest, Hartington House, Buxton. It is for ladies only, and is opened a week before Easter. The Convalescent Home, Coombe Down, Bath, also seems suitable. Droitwich, too, is celebrated for the benefit derived by rheumatic patients. The Secretary, St. John's Brine Baths, would be able to give you information.

*Up-Country Nursing Association.*

(216) Would you kindly tell me if it would be satisfactory to take up an appointment under the Up-Country Nursing Association ?—*D. S.*

Yes; the Association is a good one.

*Supplementary Training.*

(217) Having received two years' training and certificate from a general hospital, I now wish to get a post as sister, but find that a three years' certificate is necessary. I have been told that I can get a year's training at the Royal Hospital for Diseases of the Chest, that this would count as a third year's training. Would you kindly let me know if this is correct ?—*M. M.*

The matter of supplementary training is a difficult one. Some institutions require three years' continuous training, others are satisfied with a certificate from two good training schools covering the recognised three years. The City Hospital for Diseases of the Chest, Victoria Park, E., has an arrangement to give a certificate designed to meet this difficulty on the completion of a further term of training.

*Etiquette.*

(218) Having trained as a monthly nurse, would it be impudent of me to send my professional card to my former master, in whose service I had been for 15 years ?—*Nurse Alice.*

Send him your card and a little note. He should be glad to hear of your success.

*Hospital Training.*

(219) Would you be so kind as to give me some information as to training either in a French hospital or in an English hospital in France. I have had two years' fever training, and as my people are going to France, I want to know if I could complete my training there ?—*M. R.*

There is no training on the Continent to equal that of a good English hospital. You would do well to earn your three years' certificate before going abroad. The Hertford Hospital, Rue de Villiers-Levallois-Perret, Paris, might be able to give you some training in general nursing.

*Supplementary Training.*

(220) I hold a two years' certificate from a large London hospital, but I could not complete my training as my health broke down. Could you tell me if there is a hospital where a one year certificate might be gained ? Would the two certificates have an equal value with one three years' certificate ?—*M. S.*

Is it impossible to come to some arrangement with the hospital where you were trained, by which you might return and finish your course of training ? This would be best, as the three years' certificate is an advantage in some cases. There are many special hospitals where you might obtain a certificate for one year (see "The Nursing Profession: How and Where to Train"), but before deciding upon any one, if you desire employment under the Poor Law, write to the Local Government Board, and ask if the certificate you now hold, and that you intend taking, will qualify you as superintendent nurse for one of their appointments.

Could you tell me of any hospital where nurses with a two years' training are received, and where, at the end of one year's service, they receive a three years' certificate ?—*Anxious.*

See reply to *M. S.* Something of this kind is offered at the City of London Hospital for Diseases of the Chest, Victoria Park, E.

I have a three years' certificate from a provincial infirmary, and I would like to gain more experience in surgical work. Is there any institution where I could gain it without becoming a probationer again ?—*K. E. G.*

Apply to hospitals, such as the Poplar Hospital for Accidents, London, E.

*Poor Law Difficulty.*

(221) Has a master or matron of a workhouse infirmary any control over the nursing staff when there is a superintendent nurse, and can he or she search the wards ? Or has the superintendent nurse, with the doctor, full control over the nurses and nursing arrangements in the infirmary ?—*E. W.*

This friction between the nurse and master and matron of workhouses is a great difficulty in nursing under the Poor Law. The guardians make whatever arrangements they wish, so in some cases the superintendent nurse has full control of the nursing, and in others she has not, but in all except the separate infirmaries the matron is responsible for the condition of the sick as well as the general wards. It is quite as well for a nurse, before accepting an appointment, to be quite sure which rule prevails in the institution where she contemplates working.

*British Home for Incurables.*

(222) Will you kindly tell me what is your opinion of the British Home for Incurables, Streatham, what accommodation has it, and what class of patients are received ?—*H. F. S.*

This is a valuable charity designed for persons above those eligible for parochial aid. There are 76 beds.

*Drunkenness.*

(223) Will you kindly tell me of a remedy for drunkenness ? A dear friend has taken to drinking whisky to excess; if you can suggest any remedy we shall be most grateful.—*M. H.*

The best remedy is to abstain from drinking whisky at all. If your friend cannot be induced to abstain, try and persuade him or her to enter one of the Voluntary Homes for Inebriates.

*Lady Dispenser.*

(224) Can you tell me the best way for a qualified lady dispen[illegible] to obtain a post near London ?—*G. S. B.*

We are afraid that you can do nothing else but advertise u[illegible] you get what you want.

*Queen Alexandra's Nursing Service.*

(225) Where can I obtain particulars of "Queen Alexandr[illegible] Imperial Military Nursing Service" ? and to whom should a nu[illegible] apply who wishes to become a member of it ?—*A. B.*

See The Hospital *Nursing Section* of October 5th, 1901, for particulars. This service is not yet fully organised, but memb[illegible] of the existing Army and Indian and Army Nursing Rese[illegible] Services will be eligible for appointment.

*Cairo.*

(226) I should be much obliged if you could kindly give me any information about the English Nursing Home at Cairo.—*F. A. S.*

You might write to the Lady Superintendent, Victoria Nursing Home, Cairo.

*Red Cross Nurses.*

(227) I should be glad to know the headquarters of the Red Cross Nurses in England, and particulars about the training.—*Hey What.*

The headquarters of the National Society for the Aid of the Sick and Wounded in War—such is the full title of what is popularly called the Red Cross Society—is 5 York Buildings, Adelphi, W.C. The society selects nurses from the members of the Army Nursing Service, 18 Victoria Street, S.W., when required. Apply to the Hon. Secretary for particulars of training.

*Kimberley.*

(228) 1. Do you know anything of the nursing home in Kimberley, and, 2, is there more than one home in the town ?—*Inquirer.*

1. Do you mean the Kimberley and District Nursing Association, Kimberley, Notts ? If so, it is managed by a committee of nine ladies, and employs one nurse who receives £65 a year and uniform. 2. Not that we know of.

*Holt Ockley Nursing Association.*

(229) I should esteem it a favour if you will give me particulars of the Holt Ockley training.—*L. B.*

Apply the secretary, Affiliated Benefit Nursing Associations, 12 Buckingham Palace Road, S.W.

---

**Standard Books of Reference.**

"The Nursing Profession: How and Where to Train." 2s. net; post free 2s. 4d.

"Burdett's Official Nursing Directory." 3s. net; post free, 3s. 4d.

"Burdett's Hospitals and Charities." 5s.

"The Nurses' Dictionary of Medical Terms." 2s.

"Burdett's Series of Nursing Text-Books." 1s. each.

"A Handbook for Nurses." (Illustrated). 5s.

"Nursing: Its Theory and Practice." New Edition. 3s. 6d.

"Helps in Sickness and to Health." Fifteenth Thousand. 5s.

"The Physiological Feeding of Infants." 1s.

"The Physiological Nursery Chart." 1s.; post free, 1s. 3d.

"Hospital Expenditure: The Commissariat." 2s. 6d.

**All these are published by the Scientific Press, Ltd., and may be obtained through any bookseller or direct from the publishers 28 and 29 Southampton Street, London, W.C.**

### Travel Notes.

By Our Travelling Correspondent.

### XCV.—LAST DAYS IN ROME.

We have lingered long in the Eternal City and we must bring our fireside wanderings there this winter to an end to-day, though there is still so much to be visited and studied that I frankly confess I could prose on the dear old city still, probably to the weariness of my readers. There are one or two places that I should like to glance at to-day especially

### The Ghetto as it Was, and Is.

Formerly the Jews lived in comparative freedom in Trastevere, but in the middle of the sixteenth century they were strictly confined by order of Paul IV. to a portion of the city extending from the Ponte Quattro Capi to the Piazza del Pianto. This space remained their home until 1885 when it was all demolished; perhaps from a sanitary point of view this was desirable, but from the artist's and sentimentalist's ground it was grievous. The space once occupied by its varied trades and occupations now lies desolate and forsaken, but it is only since 1888 that its busy pulse has ceased to beat entirely. Let us enter the site from its far end by the Theatre of Marcellus; within its walls was built the Palazzo Savelli, where Beatrice Cenci was imprisoned—at any rate the night before her execution. This at one time was the Prussian Embassy, and Niebuhr, the historian, gives us delightful particulars of the old place in his letters. Near by, still full of memories of Beatrice, is the Palazzo Cenci.

"Just here there towers, as all the world knows, a dusky vast, irregular mass of stone and rubble that frowns on the streets beneath like a leaden storm cloud. So black it looked and frightful, frowning against the blue sky of the sweet afternoon, that for a moment I forgot what it was; one moment only, then I knew the shapeless mound was once the Theatre of Balbus; the mass built on to it and out of it was the Palace of the Cenci. On high are the grated casements whence the eyes of Beatrice once looked to see if there were any light on earth, or hope in Heaven, since she had been born in hell, and in hell must perish!"—*Ariadne.*

Passing to the Portico of Octavia there used to be the ancient Pescheria or fish market; this still flourished in 1888, but is, alas! now a thing of the past; even the antique fish slabs of imperial times were still in use, and much of the stone work in the high narrow overhanging houses must have looked down on Cæsarian Rome. Ouida, who knows the city well, thus describes market day in the Pescheria.

"It was Friday, and there was a large supply of fish still unexhausted; rosy mullets, white soles, huge cuttle fish, big spigole, sweet ombrini, black lobsters, all the fish of the Tyrrhene seas were swarming everywhere and filling all the place with salt strong pungent odours. Fish by the thousands and tens of thousands, living and dying, were crowded on the stone slabs and in the stone tanks, and on the iron hooks which jutted out between corbels and architraves, pillars and headstones. . . . She stood in the midst of the narrow way with the acrid smells and the writhing fish and the screaming people round her, and in the air the high arch restored by Septimius Severus, now daubed with bruised and peeling frescoes of the Christian church. At her side was a filthy hole, where a woman crimped a living quivering eel; above her head was a dusky unglazed window, where an old Jew turned over rusty locks and bars. She stood and looked: she who came to see the Venus of Pheidias and Praxiteles' Love."

Gregorovius, in his "Wanderjahre," gives a vivid description of the Ghetto. I can only, unfortunately, find space to quote a few lines:—

"On entering the Ghetto we see Israel, before its tents, in full restless labour and activity. The people sit in their doorways, or outside in the streets, which receive hardly more light than the damp and gloomy chambers, and grub amid their old trumpery or patch and sew diligently. The whole world seems to be lying about in countless rags and scraps, as Jewish plunder. The fragments lie in heaps before the doors; they are of every kind of colour—gold fringes, scraps of silk brocade, bits of velvet, red patches, blue patches, orange, yellow, black and white, torn, old, slashed and tattered pieces, large and small. I never saw such varied rubbish. The Jews might mend up all creation, and patch the whole world as gaily as harlequin's coat. . . . Here sit the daughters of Zion on these heaps, and sew all that is capable of being sewn. Great is their boasted skill in all works of mending, darning and fine drawing, and it is said that even the most formidable rent in any old drapery or garment whatsoever becomes invisible under the hands of these modern Arachnes. . . . I have often seen with a feeling of pain the pale, stooping, starving figures laboriously plying the needle, men as well as women, girls and children. Misery stares forth from the tangled hair and complains silently in the yellow brown faces, and no beauty of feature recalls the countenance of Rachel, Leah, or Miriam—only sometimes a glance from a deep sunk, piercing black eye that looks up from its needle and rags and seems to say 'From the daughter of Zion all her beauty is departed.'"

### The Villa Borghese.

I should like you to leave Rome with more cheerful recollections than those of the Ghetto, so let me beg you to be a frequent visitor to the lovely Borghese Gardens in the spring. These noble pleasure grounds have been always open to the public, since their foundation early in the sixteenth century. They lie just outside the Porta del Popolo. In the early part of the year the ground in places is carpeted with anemones and violets and a peculiarly fine kind of daisy, the same as Burns's "wee modest crimson-tipped flower," only very much larger and on a stalk some six or seven inches long. The familiar name of them is "Campagna daisy."

And now farewell to Rome.

---

### TRAVEL NOTES AND QUERIES.

Rules in Regard to Correspondence for this Section.—All questioners must use a pseudonym for publication, but the communication must also bear the writer's own name and address as well, which will be regarded as confidential. All such communications to be addressed "Travel Editor, 'Nursing Section of *The Hospital*,' 28 Southampton Street, Strand." No charge will be made for inserting and answering questions in the inquiry column, and all will be answered in rotation as space permits. If an answer by letter is required, a stamped and addressed envelope must be enclosed, together with 2s. 6d., which fee will be devoted to the objects of "The Hospital" Convalescent Fund. Any inquiries reaching the office after Monday cannot be answered in "The Hospital" of the current week.

Cromer in May (Hygeine).—Cromer is an expensive place, but write to the following addresses and ask for terms, stating that they must be reasonable:—Mrs. Newman, Beach House Boarding Establishment; Manageress, Surrey House Boarding House; and Mrs. Arthur Vicary, Westward Ho! If all these prove too dear, you might try lodgings, which used to be very good. Leave your luggage at the station and look about; you can generally get some addresses at the confectioners. I must warn you again that nothing is very cheap in Cromer.

France for Good French Accent (Nurse S.).—I could help you better if I knew more. You are so vague. Do you mean suburbs of Paris, or would you like such a place as Fontainebleau or Chartres? There are some nice cheap little places on the Seine between Rouen and Havre, notably Caudebec. There is a prejudice in favour of Tours as a place in which to acquire the purest accent. The cheapest route to Paris is *viâ* London, Southampton, and Havre, second return £2 0s. 8d.; *viâ* Newhaven and Dieppe, £2 2s. 3d., third-class return £1 3s. 3d. Can you speak French fluently, if so, lodgings may be had, but for that a knowledge of the language is necessary. Write me more fully, who your party consists of, what money you can spend each, and how long you wish to stay; whether you must be in Paris, and, above all, whether you can *talk.* I should rather recommend you Rouen and the banks of the Seine.

## Figures and Tables

**Fig. 31. f1:**
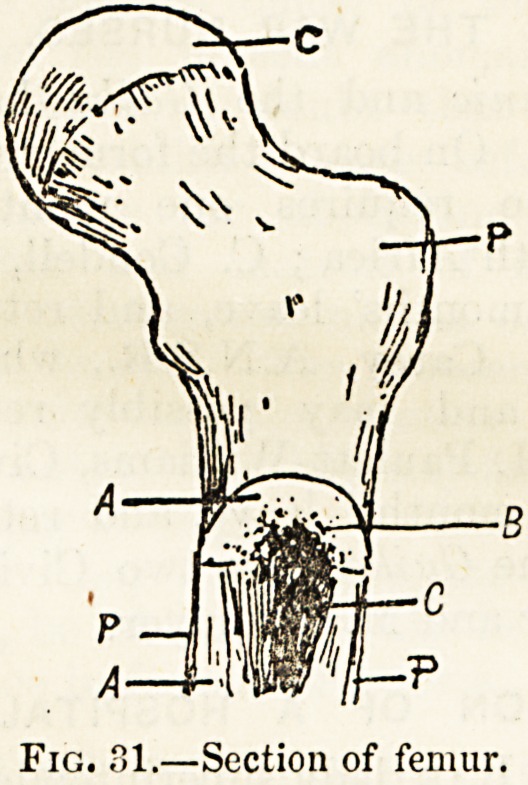


**Fig. 32. f2:**
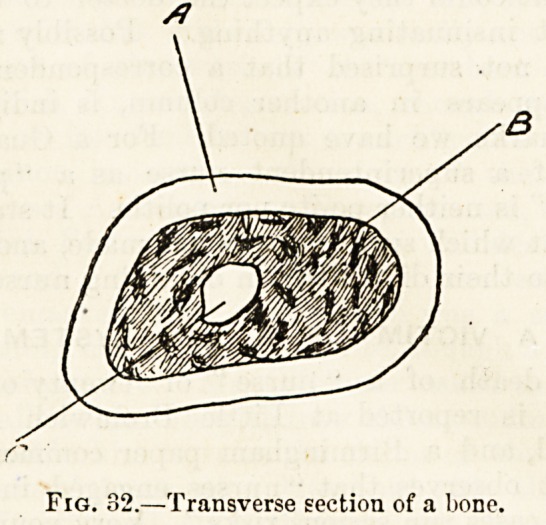


**Fig. 33. f3:**